# Cutaneous squamous cell carcinoma staging may influence management in users: A survey study

**DOI:** 10.1002/cam4.4426

**Published:** 2021-11-18

**Authors:** Vishal A. Patel, Catherine McCullum, Andrew D. Sparks, Chrysalyne D. Schmults, Sarah T Arron, Anokhi Jambusaria‐Pahlajani

**Affiliations:** ^1^ Department of Dermatology George Washington School of Medicine and Health Sciences Washington District of Columbia USA; ^2^ Department of Dermatology Brigham and Women's Hospital Harvard Medical School Boston Massachusetts USA; ^3^ Sarah Arron Medical Professional Corporation San Mateo California USA; ^4^ Division of Dermatology Department of Internal Medicine The University of Texas at Austin Dell Medical School Austin Texas USA

**Keywords:** American Joint Committee on Cancer Staging System, Brigham and Women Staging System, depth of tumor, high‐risk cutaneous squamous cell carcinoma, high‐risk tumor features, histologic differentiation, immunosuppression, perineural invasion, skin cancer, staging criteria, tumor diameter, tumor location

## Abstract

**Purpose:**

This study aims to determine whether there is consensus regarding staging and management of cutaneous squamous cell carcinoma (CSCC) across the various specialties that manage this disease.

**Materials and Methods:**

A survey regarding CSCC high‐risk features, staging, and management was created and emailed to cutaneous oncology experts including dermatology, head and neck surgery/surgical oncology, radiation oncology, and medical oncology.

**Results:**

One hundred fifty‐six (46%) of 357 invited physicians completed the survey. Depth of invasion (92%), perineural invasion (99%), histologic differentiation (85%), and patient immunosuppression (90%) achieved consensus (>80%) as high‐risk features of CSCC. Dermatologists were more likely to also choose clinical tumor diameter (79% vs. 54%) and histology (99% vs. 66%) as a high‐risk feature. Dermatologists were also more likely to utilize the Brigham and Women's Hospital (BWH) staging system alone or in conjunction with American Joint Committee on Cancer (AJCC) (71%), whereas other cancer specialists (OCS) tend to use only AJCC (71%). Respondents considered AJCC T3 and higher (90%) and BWH T2b and higher (100%) to be high risk and when they consider radiologic imaging, sentinel lymph node biopsy, post‐operative radiation therapy, and increased follow‐up. Notably, a large number of respondents do not use staging systems or tumor stage to determine treatment options beyond surgery in high‐risk CSCC.

**Conclusion:**

This survey study highlights areas of consensus and differences regarding the definition of high‐risk features of CSCC, staging approaches, and management patterns between dermatologists and OCS. High‐risk CSCC is defined as, but not limited to, BWH T2b and higher and AJCC T3 and higher, and these thresholds can be used to identify cases for which treatment beyond surgery may be considered. Dermatologists are more likely to utilize BWH staging, likely because BWH validation studies showing advantages over AJCC were published in dermatology journals and discussed at dermatology meetings. Additional data are necessary to develop a comprehensive risk‐based management approach for CSCC.

## Graphical abstract

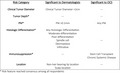

## 1 INTRODUCTION

Cutaneous squamous cell carcinoma (CSCC) is a rising epidemic, and while most tumors having an excellent prognosis, 4%–6% of tumors metastasize and 1.5%–2% lead to death.^1^, ^2^, ^3^, ^4^ Over the past several years, multiple CSCC staging systems and guidelines have been developed that vary in tumor assessment, including the AJCC 8^th^ Edition (AJCC), Brigham and Women's Hospital tumor staging system (BWH), University Hospital Tubingen (UHT) system, and National Comprehensive Cancer Network (NCCN) guidelines.^5^ With no universal accepted definition of high‐risk CSCC (HRCSCC), clear management guidelines are lacking. Point estimates of risk for recurrence and metastasis by tumor stage have varied between studies.^6^, ^7^, ^8^ Subsequently, studies have found notable differences regarding the perioperative management of HRCSCC among Mohs surgeons, radiation oncologists, and head and neck surgeons.^9^, ^10^ Due to a paucity of data regarding the utility of nodal staging and adjuvant therapy, HRCSCC treatment is inconsistent and based on anecdotal experience.

This study aims to assess how physicians across the fields of dermatology, head and neck surgery/surgical oncology, radiation oncology, and medical oncology (latter three groups categorized as Other Cancer Specialists [OCS]) define HRCSCC and approach high risk, advanced, and/or metastatic CSCC. We hypothesized that there is variability between dermatologists and OCS with regard to HRCSCC definition, staging utilization, and management. Areas of consensus and areas needing clarification with future studies are identified.

## 2 METHODS

A 25‐question “Cutaneous SCC Staging Survey” (Figure S1) was developed by four of the authors (VAP, AJP, CDS, and STA) encompassing the management of HRCSCC. A list of dermatologists from the International Immunosuppression and Transplant Skin Cancer Collaborative and the American College of Mohs Surgery MohsAIQ registry, considered experts in cutaneous oncology, was created.^11^ These individuals were contacted by email for OCS colleague referrals who frequently manage HRCSCC patients. Using these recommendations, a group of dermatologists and OCS were invited to participate.

Invited participants were emailed a personalized REDCap weblink to the survey and sent two reminders over 4 weeks. Incomplete surveys were excluded. The study was approved by the University of Texas at Austin Dell Medical School institutional review board.

### 2.1 Statistical analysis

Descriptive summary statistics for survey responses were reported as frequency (percentage). Consensus was determined *a priori* at 80% or more of respondents in agreement. Comparisons between dermatologists and OCS were analyzed using Chi‐square test (or Fisher's exact test for a cell‐count ≤5). SAS version 9.4 was used for statistical analysis (SAS Institute Inc.). A two‐sided *p*‐value ≤0.05 was considered statistically significant.

## 3 RESULTS

A total of 337 physicians (*n* = 163, 48% dermatologists and *n* = 174, 52% OCS) were invited to participate in the study. One hundred and fifty‐six (46%) physicians completed the survey, of which 89 (57%) were dermatologists and 67 were (43%) OCS. Respondent characteristics, including specialty, number of years practicing, practice setting, and self‐reported number of HRCSCCs managed in the previous 12 months are listed in Table 1.

**TABLE 1:** Baseline demographics of survey respondents

	Number of responses (% of cohort)	Dermatologists	Other cancer specialists (OCS)
**Total respondents**	156	89 (57.05%)	67 (42.95%)
**Specialty**			
Med Dermatology/Dermatology Oncology	14 (8.97%)		
Mohs	75 (48.08%)		
Medical Oncology	15 (9.62%)		
Surgical Oncology	1 (0.64%)		
ENT/Head and Neck Surgery	27 (17.31%)		
Plastic Surgery	3 (1.92%)		
Radiation Oncology	21 (13.46%)		
**Region**			
USA	148 (94.87%)	83 (93.26%)	65 (97.01%)
UK	5 (3.21%)	5 (5.62%)	0
Canada	2 (1.28%)	1 (1.12%)	1 (1.49%)
Australia/New Zealand	1 (0.64%)	0	1 (1.49%)
Europe	0	0	0
**Number of HRCSCC treated in the past 12 months**			
0	0	0	0
1–10	25 (16.03%)	12 (13.48%)	13 (19.40%)
11–25	75 (48.08%)	45 (50.56%)	30 (44.78%)
26–50	32 (20.51%)	14 (15.73%)	18 (26.87%)
>50	24 (15.38%)	18 (20.22%)	6 (9.95%)
**Number of years in practice (post‐residency)**			
0–5	46 (29.49%)	27 (30.34%)	19 (28.36%)
6–10	45 (28.85%)	27 (30.34%	18 (26.87%)
11–20	36 (23.08%)	19 (21.37%)	17 (25.37%)
>20	29 (18.59%)	16 (17.98%)	13 (19.40%)
**Practice environment**			
Academics	136 (87.18%)	72 (80.90%)	64 (95.52%)
Private practice/closed multi‐specialty	31 (19.87%)	22 (24.72%)	5 (7.46%)
VA	7 (4.49%)	6 (6.74%)	1 (1.49%)
Open multi‐specialty group	1 (0.64%)	1 (1.12%)	0

Abbreviation: HRSCC, high‐risk squamous cell carcinoma.

### 3.1 High‐risk features

Table S1 (ST1) lists the percentage of respondents selecting a feature as high risk.

#### 3.1.1 Areas of consensus

**FIGURE 1:**
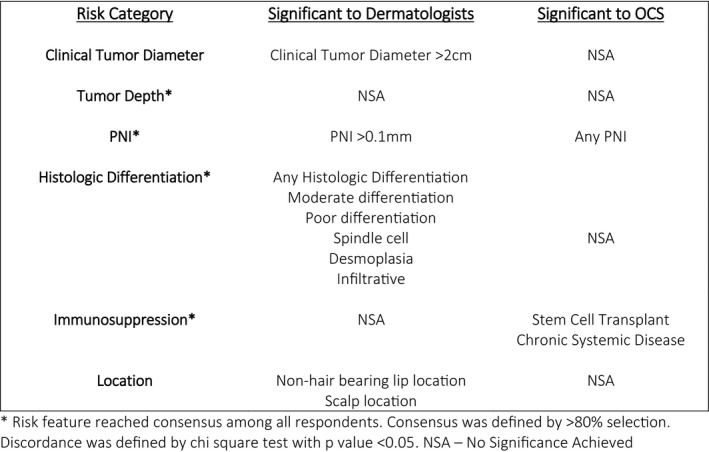
Consensus and discordance of risk features among respondents

Features selected by ≥80% of respondents included: depth of invasion (DI) (*n* = 143, 92%), perineural invasion (PNI) (*n* = 154, 99%), poor histologic differentiation (HD) (*n* = 132, 85%), and immunosuppression (*n* = 141, 90%) (Figure 1).

#### 3.1.2 Areas of divergence

Clinical tumor diameter (CTD) and HD were significantly more likely to be chosen by dermatologists as high risk compared to OCS (79% vs. 54%; *p *= 0.001 and 99% vs. 66%; *p *< 0.001, respectively) (Figure 1). Dermatologists considered >2 cm diameter, moderate differentiation, poor differentiation, spindle cell, desmoplasia, and infiltration to be high risk significantly more often than OCS (*p *< 0.001, *p *= 0.0005, <0.001, 0.001, 0.021, 0.011, respectively). OCS were more likely to select any PNI as high risk (40% vs. 17%, *p* < 0.001), while dermatologists more often designated PNI of a large caliber nerve (≥0.1 mm diameter) as high risk (92% vs. 66%, *p *< 0.001). While a majority of respondents considered patients with solid organ transplantation (SOTR) (*n* = 94; 60.3%) and hematologic malignancy (*n* = 113; 72.4%) at high risk for poor outcome, OCS considered stem cell transplant (SCT), and chronic systemic disease as high risk more often than dermatologists (*p *= 0.0285, 0.001, respectively).

### 3.2 Staging system utilization

Most dermatologists (89%) and OCS (93%) currently stage CSCC tumors. OCS preferred to stage all tumors (*n* = 48, 72%), while dermatologists tended to stage only high‐risk tumors (*n* = 59, 66%, *p*‐value <0.001). AJCC and BWH were the predominate staging systems utilized. Dermatologists (71%) were likely to utilize the Brigham and Women's Hospital (BWH) staging system alone or in conjunction with American Joint Committee on Cancer (AJCC), whereas OCS (71%) used only AJCC. Among those using BWH staging, there was broad consensus that BWH T2b and higher CSCC are high risk (100% of dermatologists, 100% of OCS). Among those using AJCC staging, there was broad consensus that AJCC T3 and higher CSCC are high risk (96% of dermatologists, 93% of OCS). (Table 2).

**TABLE 2:** Tumor staging utilization and management patterns

	Dermatologists	OCS	*p*‐Value
**Do you currently stage CSCC tumors that present to your practice?**	**(*n* = 89)**	**(*n* = 67)**	
Yes No No response	79 (89%) 8 (9%) 2 (2%)	62 (93%) 5 (8%) 0 (0%)	<0.001
**If you do stage tumors, which tumors do you stage?**	**(*n* = 89)**	**(*n* = 67)**	
All tumors Suspected high risk Other	19 (24%) 59 (75%) 1 (1%)	48 (77%) 14 (23%) 0 (0%)	<0.001
**If you do stage tumors, which staging system do you use?**	**(*n* = 89)**	**(*n* = 67)**	
AJCC BWH Both Other	12 (13%) 26 (29%) 37 (42%) 14 (16%)	44 (71%) 1 (2%) 12 (19%) 10 (16%)	<0.001*
**If you utilize staging criteria to identify HRCSCC, which stages do you consider as high risk in AJCC?**	**(*n* = 49)**	**(*n* = 56)**	
T1 and higher T2 and higher T3 and higher T4 and higher	0 (0.0%) 25 (51%) 22 (45%) 1 (2%)	4 (7%) 20 (36%) 28 (50%) 2 (4%)	N/A
**If you utilize staging criteria to identify HRCSCC, which stages do you consider as high risk in BWH‐T?**	**(*n* = 63)**	**(*n* = 13)**	
T1 and higher T2a and higher T2b and higher T3 and higher	0 (0%) 7 (11%) 56 (89%) 0 (0%)	0 (0%) 1 (8%) 12 (92%) 0 (0%)	N/A
**If you use pre or post‐operative radiologic imaging to detect subclinical lymph node metastasis or distant metastasis in a patient with HRCSCC, what staging do you use to determine imaging?**	**(*n* = 89)**	**(*n* = 67)**	
AJCC BWH Both Other	6 (6.7%) 34 (38.2%) 24 (27.0%) 25 (28.1%)	34 (50.7%) 4 (6.0%) 6 (9.0%) 23 (34.3%)	<0.001
**For which AJCC stage do you consider radiologic imaging to detect subclinical lymph node metastasis or distant metastasis?**	**(*n* = 28)**	**(*n* = 40)**	
T1 and higher T2 and higher T3 and higher T4 and higher	0 (0.0%) 11 (36.7%) 16 (53.3%) 1 (3.3%)	0 (0.0%) 16 (40.0%) 22 (55.0%) 0 (0.0%)	N/A
**For which BWH stage do you consider radiologic imaging to detect subclinical lymph node metastasis or distant metastasis?**	**(*n* = 58)**	**(*n *= 10)**	
T1 and higher T2a and higher T2b and higher T3 and higher	0 (0.0%) 3 (5.2%) 54 (93.1%) 1 (1.7%)	0 (0.0%) 2 (20.0%) 8 (80.0%) 0 (0.0%)	N/A
**If you use or recommend SLNB to search for sub‐clinical lymph node metastasis in a patient with HRCSCC, what staging system do you use to determine SLNB?**	**(*n* = 89)**	**(*n* = 67)**	
AJCC BWH Both Other	5 (5.6%) 23 (25.8%) 15 (16.9%) 46 (51.7%)	18 (26.9%) 5 (7.5%) 3 (4.5%) 41 (61.2%)	<0.001
**For which AJCC stage do you consider SLNB to detect subclinical lymph node metastasis or distant metastasis?**	**(*n* = 20)**	**(*n* = 21)**	
T1 and higher T2 and higher T3 and higher T4 and higher	0 (0%) 3 (15%) 17 (85%) 0 (0%)	0 (0%) 7 (33%) 13 (62%) 0 (0%)	N/A
**For which BWH stage do you consider SLNB to detect subclinical lymph node metastasis or distant metastasis?**	**(*n* = 38)**	**(*n* = 8)**	
T1 and higher T2a and higher T2b and higher T3 and higher	0 (0%) 0 (0%) 36 (95%) 2 (5%)	0 (0.0%) 3 (38%) 5 (62%) 0 (0%)	N/A
**If you use or recommend post‐operative ART for a patient with HRCSCC, what staging system do you use to determine post‐operative ART?**	**(*n* = 89)**	**(*n* = 67)**	
AJCC BWH Both Other	6 (7%) 29 (33%) 27 (30%) 27 (30%)	36 (54%) 4 (6%) 7 (10%) 20 (30%)	<0.001
**For which AJCC stage do you consider post‐operative ART for a patient with HRCSCC?**	**(*n* = 33)**	**(*n* = 43)**	
T1 and higher T2 and higher T3 and higher T4 and higher	0 (0%) 7 (21%) 24 (73%) 1 (3%)	0 (0%) 12 (8%) 28 (65%) 2 (5%)	N/A
**For which BWH stage do you consider post‐operative ART for a patient with HRCSCC?**	**(*n* = 66)**	**(*n* = 11)**	
T1 and higher T2a and higher T2b and higher T3 and higher	0 (0%) 2 (4%) 51 (91%) 3 (5%)	0 (0%) 1 (9%) 10 (91%) 0 (0%)	N/A
**If you use or recommend post‐operative adjuvant systemic therapy for a patient with HRCSCC, what staging system do you use to determine adjuvant therapy?**	**(*n *= 89)**	**(*n* = 67)**	
AJCC BWH Both Other	2 (2%) 10 (11%) 7 (8%) 70 (79%)	15 (22%) 0 0%) 3 (4%) 49 (73%)	<0.001
**For which AJCC stage do you consider post‐operative adjuvant systemic therapy for a patient with HRCSCC?**	**(*n* = 9)**	**(*n* = 18)**	
T1 and higher T2 and higher T3 and higher T4 and higher	0 (0.0%) 1 (11%) 5 (56%) 2 (22%)	0 (0.0%) 3 (17%) 8 (44%) 5 (28%)	N/A
**For which BWH stage do you consider post‐operative adjuvant systemic therapy for a patient with HRCSCC?**	**(*n* = 17)**	**(*n *= 3)**	
T1 and higher T2a and higher T2b and higher T3 and higher	0 (0%) 0 (0%) 10 (59%) 7 (41%)	0 (0%) 0 (0%) 2 (67%) 0 (0%)	N/A
**If you do recommend increased follow‐up frequency for a skin and lymph node evaluation for a patient with HRCSCC, what staging system do you use to recommend follow‐up?**	**(*n* = 89)**	**(*n* = 67)**	
AJCC BWH Both Other	6 (7%) 29 (33%) 26 (29%) 28 (31%)	25 (37%) 3 (5%) 5 (7%) 34 (51%)	<0.001
**For which AJCC stage do you consider increased follow‐up for a patient with HRCSCC?**	**(*n* = 32)**	**(*n* = 30)**	
T1 and higher T2 and higher T3 and higher T4 and higher	0 (0%) 10 (31%) 21 (66%) 0 (0%)	2 (7%) 8 (27%) 20 (67%) 0 (0%)	N/A
**For which BWH stage do you consider increased follow‐up for a patient with HRCSCC?**	**(*n *= 55)**	**(*n* = 8)**	
T1 and higher T2a and higher T2b and higher T3 and higher	0 (0%) 3 (5%) 51 (93%) 0 (0%)	0 (0%) 2 (25%) 6 (75%) 0 (0%)	N/A

* *p*‐value reflects only comparison between those using exclusively AJCC or exclusively BWH‐T.

### 3.3 Disease management

A large number of respondents reported they do not use staging systems to help consider whether to perform radiologic imaging, SLNB, post‐operative radiation therapy (PORT), adjuvant systemic therapy, or increased follow‐up in HRCSCC (31%, 56%, 30%, 76%, 40%, respectively). However, of those utilizing BWH staging, there was consensus that BWH T2b cases could be considered for radiologic imaging (98%, 100%), SLNB (95%, 100%), PORT (95%, 100%), and increased follow‐up (98%, 100%) by dermatologists and OCS, respectively. For those utilizing AJCC staging, there was consensus that AJCC T3 cases could be considered for radiologic imaging (90%, 95%), SLNB (100%, 95%), PORT (94%, 93%), and increased follow‐up (97%, 100%) by dermatologists and OCS, respectively. (Table 2).

## 4 DISCUSSION

Accurate identification of HRCSCC helps to ensure we develop robust staging systems, identify those at high risk of poor outcomes, and provide precise treatments based on risk. The purpose of this study was to provide a multidisciplinary perspective regarding HRCSCC risk assessment and management. PNI, DI, HD, and immunosuppression reached consensus as high‐risk factors by all experts. PNI ≥0.1 mm diameter, CTD (including >2 cm tumor diameter), and HD (including moderate differentiation, poor differentiation, spindle cell, desmoplasia, and infiltration) were more likely to be chosen by dermatologists than OCS. Our study demonstrates consensus in line with recent reports validating high‐risk features and poor outcomes but also highlights the variability in definitions between experts.^9^, ^10^ The impact of such variability can be seen in the two current leading clinical trials evaluating adjuvant immunotherapy in HRCSCC; the inclusion criteria are different for both trials and do not follow either AJCC or BWH staging.^12^, ^13^ Results of these trials will be variable, making it difficult for clinicians or the NCCN to draw generalizable conclusions regarding adjuvant therapy.

Nearly all respondents selected PNI and 81% specified that nerve diameter >0.1 mm was a significant cut‐off. However, OCS were more likely to select any PNI as a risk factor, while dermatologists were more likely to specify PNI ≥0.1 mm diameter as high risk. This difference is critical as there is notable evidence in the dermatology literature that PNI of nerves <0.1 mm in diameter in the absence of other risk factors does not impact prognosis.^14^, ^15^ The lack of widespread discussion in the oncology literature may explain this variability. Dermatologists and Mohs surgeons also dedicate a large portion of their practice to HRCSCC, while most OCS are unlikely to have HRCSCC focus alone. While current staging and practice guidelines include PNI as a risk factor; ambiguity remains regarding management of the range of PNI and the variability of understanding true risk may lead to overtreatment in patients with small‐caliber PNI.^10^, ^16^

Immunosuppression is another feature with variable response. While clinical factors are typically not included in staging systems, there was a clear consensus of immunosuppression, and specifically SOTRs, as an important risk factor. This is unsurprising given that it is well known that SOTR who develop CSCC have worse outcomes than immunocompetent patients.^17^, ^18^ However, the identified risk from other immunosuppressive conditions was more variable between experts with OCS more likely to consider stem cell transplant and chronic systemic diseases as high risk compared to dermatologists. This is notable considering that evidence of increased risk is minimal in these conditions.^19^ The presence of immunosuppression needs clarity in regard to how to best incorporate this information with staging systems to make management decisions.

Uncertainty remains regarding what staging systems are best for risk assessment and management decisions for CSCC. Dermatologists utilize BWH staging more frequently than OCS, either on its own or in conjunction with AJCC, while OCS generally preferred AJCC. This is likely due to BWH being developed by dermatologists and is well discussed at dermatology conferences and in the dermatology literature.^6^, ^7^ OCS are likely accustomed to AJCC staging for other cancers and may thus prefer it. BWH has been shown to be superior to AJCC and many of the BWH risk factors were incorporated into the update from the seventh to eighth edition of AJCC, but with only 40% of respondents utilizing AJCC, it clearly needs further refinement before it is utilized widely.^11^, ^20^ Furthermore, 15% of respondents noted utilizing neither system highlighting a clear practice gap in the staging of CSCC.

There was consensus among those who used AJCC and BWH that tumor stage ≥AJCC T3 or ≥BWH T2b should be considered high‐risk and radiologic imaging, SLNB, adjuvant radiation therapy, and increased follow‐up would be considered in these tumors. This threshold to consider adjuvant therapy is not surprising given the number of studies that have shown high poor outcomes rates in these tumors.^21^, ^22^ However, the exact clinical scenarios and management options that should be employed in these patients are uncertain. Specifically, not all BWH T2b and AJCCT3 cases portend sufficient risk to warrant adjuvant therapy blindly, and data and guidelines vary regarding recurrence risk and management of this CSCC subset. Furthermore, there are a number of AJCC T2 and BWH T2a tumors that metastasize and lead to death.^19^ This may explain why 43% of AJCC users denote T2 to be high risk and that AJCC T2/T3 tumors have a difficult overlap to discern between. The NCCN also recently created a new “very high risk” designation to unify management of a subset of tumors that have poor outcomes. (Table 3).

**TABLE 3:** CSCC staging and management systems

AJCC				
T stage		Risk Factors (Head and Neck Only)		
T1		Tumor diameter <2 cm		
T2		Tumor diameter ≥2 cm and <4 cm in greatest dimension		
T3		Tumor diameter ≥4 cm, or minor bone erosion, or perineural invasion, or deep invasion		
T4		Tumor with gross cortical bone/marrow invasion		
BWH				
T stage		Risk factors		
T1		No high‐risk factors		
T2a		1 High‐risk factor		
T2b		2–3 High‐risk factors		
T3		≥4 High‐risk factors		
*High‐risk factors: Tumor diameter 2 cm, invasion beyond subcutaneous fat, poorly differentiated, and perineural invasion				
NCCN Version 1.2021				
	Low Risk		High Risk	Very High Risk
Location/size	Trunk, extremities <2 cm		Trunk, extremities ≥2 cm Head, neck, hands, feet, pretibial, and anogenital (any size)	>4 cm any location
Borders	Well‐defined		Poorly defined	Poorly defined
Primary vs. recurrent	Primary		Recurrent	Recurrent
Immunosuppression	(−)		(+)	(+)
Site of prior radiation therapy or chronic inflammatory process	(−)		(+)	(+)
Rapidly growing tumor	(−)		(+)	(+)
Neurologic symptoms	(−)		(+)	(+)
Degree of differentiation	Well or moderately differentiated		Poorly differentiated	Poorly differentiated
Acantholytic, adenosquamous, desmoplastic, or metaplastic subtypes	(−)		(+)	(+)
Depth, thickness, or level of invasion	≤6 mm and no invasion beyond subcutaneous fat		>6 mm or invasion beyond subcutaneous fat	>6 mm or invasion beyond subcutaneous fat
Perin‐eural involvement	(−)		(+)	Tumor cells within the nerve sheath of a nerve lying deeper than the dermis or measuring >0.1 mm
Lymphatic or vascular involvement	(−)		(−)	(+)

More concerning is that a large number of respondents are not using either system to drive their decision to perform radiologic imaging, SLNB, adjuvant radiation therapy, adjuvant systemic therapy, or increased follow‐up. There is no clear consensus about how to utilize T staging to drive management despite evidence of the benefit of additional interventions in HRCSCC.^23^, ^24^ Uncertainty is expected given the inconsistent risk estimates by T stage.^19^, ^20^ Furthermore, the scarcity of staging system implementation in clinical practice may be compounded by the high incidence of CSCC, together with the T stage heterogeneity, as well as the potential lack of concern or knowledge about this tumor compared with melanoma. The underutilization of consistent and concrete guidelines signifies a major shortcoming in the care of patients with CSCC, especially HRCSCC, and needs urgent rectifying with accurate T‐stage risk estimates and evidence‐based treatment modalities by disease stage.

A strength of this study is that we surveyed experts in cutaneous oncology who treat a high number of HRCSCC, which allowed answers to be based on collective experiences. A limitation is that the panelists were identified from mainly academic tertiary referral centers and thus results may not be generalizable to all physician practice patterns. The survey was developed based on accepted literature evidence and current staging, but given the lack of complete understanding of CSCC, some answer choices in the survey may have been omitted. In these cases, many experts entered text that was not categorizable and thus not analyzed.

The results of this survey illustrate areas of consensus and equipoise between multispecialty experts that manage high risk and advanced CSCC. While there are many areas of agreement, there is significant room for communication and education. But most importantly, these results highlight the need for multidisciplinary consensus building and clinical trials to establish evidence‐based criteria for risk stratification and management of HRCSCC.

## CONFLICTS OF INTEREST

None.

## AUTHORS CONTRIBUTIONS

Patel VA and Jambusaria‐Pahlajani A involved in study conception and design. Patel VA and McCullum C carried out data collection. Patel VA, McCullum C, Sparks AD, Schmults CD, Arron ST, and Jambusaria‐Pahlajani A involved in analysis and interpretation of results. Patel VA, McCullum C, Schmults CD, Arron ST, and Jambusaria‐Pahlajani A carried out draft manuscript preparation. All authors reviewed the results and approved the final version of the manuscript.

## ETHICAL STATEMENT

The research was conducted in accordance with the principles embodied in the Declaration of Helsinki and in accordance with local statutory requirements. All participants gave written informed consent to participate in the study.

## Supporting information

Fig S1

Table S1

## References

[1] Rogers HW , Weinstock MA , Feldman SR , et al. Incidence estimate of nonmelanoma skin cancer (keratinocyte carcinomas) in the US population, 2012. JAMA Dermatol. 2015;151(10):1081‐1086. 25928283 10.1001/jamadermatol.2015.1187

[2] Brantsch KD , Meisner C , Schönfisch B , et al. Analysis of risk factors determining prognosis of cutaneous squamous cell carcinoma: a prospective study. Lancet Oncol. 2008;9(8):713‐720. 18617440 10.1016/S1470-2045(08)70178-5

[3] Schmults CD , Karia PS , Carter JB , et al. Factors predictive of recurrence and death from cutaneous squamous cell carcinoma: a 10‐year, single‐institution cohort study. JAMA Dermatol. 2013;149(5):541‐547. 23677079 10.1001/jamadermatol.2013.2139

[4] Czarnecki D , Staples M , Mar A , et al. Metastases from squamous cell carcinoma of the skin in southern Australia. Dermatology. 1994;189:52‐54. 10.1159/000246783 8003787

[5] Roscher I , et al. Validating 4 staging systems for cutaneous squamous cell carcinoma using population‐based data: a nested case‐control study. JAMA Dermatol. 2018;154(4):428‐434. doi:10.1001/jamadermatol.2017.6428 29516080 PMC5876840

[6] Karia PS , Jambusaria‐Pahlajani A , Harrington DP , et al. Evaluation of American Joint Committee on Cancer, International Union Against Cancer, and Brigham and Women's Hospital tumor staging for cutaneous squamous cell carcinoma. J Clin Oncol. 2014;32:327‐334. 24366933 10.1200/JCO.2012.48.5326 PMC3897257

[7] Marrazzo G , Zitelli J , Broadland D . Clinical outcomes in high‐risk squamous cell carcinoma patients treated with Mohs micrographic surgery alone. J Am Acad Dermatol. 2019;80:633‐638. 30244064 10.1016/j.jaad.2018.09.015

[8] Tschetter AJ , Campoli MR , Zitelli JA , et al. Long‐term clinical outcomes of patients with invasive cutaneous squamous cell carcinoma treated with Mohs micrographic surgery: a 5‐year, multicenter, prospective cohort study. J Am Acad Dermatol. 2020;82:139‐148. 31279037 10.1016/j.jaad.2019.06.1303

[9] Thompson AK , Kelley BF , Prokop LJ , et al. Risk factors for cutaneous squamous cell carcinoma recurrence, metastasis, and disease‐specific death: a systematic review and meta‐analysis. JAMA Dermatol. 2016;152(4):419‐428. 26762219 10.1001/jamadermatol.2015.4994 PMC4833641

[10] Que SKT , Zwald FO , Schmults CD . Cutaneous squamous cell carcinoma: Incidence, risk factors, diagnosis, and staging. J Am Acad Dermatol. 2018;78(2):237‐247. 29332704 10.1016/j.jaad.2017.08.059

[11] Crow L , Jambusaria‐Pahlajani A , Chung CL , et al. Initial skin cancer screening for solid organ transplant recipients in the United States: delphi method development of expert consensus guidelines. Transpl Int. 2019;32(12):1268‐1276. 31502728 10.1111/tri.13520

[12] Clinicaltrials.gov . https://clinicaltrials.gov/ct2/show/NCT03833167?term=pembrolizumab&cond=Cutaneous+Squamous+Cell+Carcinoma&draw=2&rank=5

[13] Clinicaltrials.gov . https://clinicaltrials.gov/ct2/show/NCT03969004?term=cemiplimab&cond=adjuvant&draw=2&rank=1

[14] Carter JB , Johnson MM , Chua TL , et al. Outcomes of primary cutaneous squamous cell carcinoma with perineural invasion: an 11‐year cohort study. JAMA Dermatol. 2013;149(1):35‐41. 23324754 10.1001/jamadermatol.2013.746

[15] Ross S , Miller Whalen B , Elenitsas D , Xu D , Troxel D , Schmults D . Diameter of involved nerves predicts outcomes in cutaneous squamous cell carcinoma with perineural invasion: an investigator‐blinded retrospective cohort study. Dermatol Surg. 2009;35(12):1859‐1866. doi:10.1111/j.1524-4725.2009.01354.x 19889009

[16] Jambusaria‐Pahlajani A , Kanetsky PA , Karia PS , et al. Evaluation of AJCC tumor staging for cutaneous squamous cell carcinoma and a proposed alternative tumor staging system. JAMA Dermatol. 2013;149(4):402‐410. 23325457 10.1001/jamadermatol.2013.2456

[17] Lanz J , Bouwes Bavinck JN , Westhuis M , et al. Aggressive squamous cell carcinoma in organ transplant recipients. JAMA Dermatol. 2019;155(1):66‐71. doi:10.1001/jamadermatol.2018.4406 30516812 PMC6439577

[18] Cheng JY , Li F‐Y , Ko CJ , Colegio OR . Cutaneous squamous cell carcinomas in solid organ transplant recipients compared with immunocompetent patients. JAMA Dermatol. 2018;154(1):60‐66. doi:10.1001/jamadermatol.2017.4506 29167858 PMC5833573

[19] Omland SH , Gniadecki R , Hædersdal M , et al. Skin cancer risk in hematopoietic stem‐cell transplant recipients compared with background population and renal transplant recipients: a population‐based cohort study. JAMA Dermatol. 2016;152(2):177‐183. 26454261 10.1001/jamadermatol.2015.3902

[20] Ruiz ES , Karia PS , Besaw R , Schmults CD . Performance of the American Joint Committee on cancer staging manual, 8th edition vs the brigham and women’s hospital tumor classification system for cutaneous squamous cell carcinoma. JAMA Dermatol. 2019;155(7):819‐825. 30969315 10.1001/jamadermatol.2019.0032 PMC6583833

[21] Cañueto J , Burguillo J , Moyano‐Bueno D , et al. Comparing the eighth and the seventh editions of the American Joint Committee on Cancer staging system and the Brigham and Women's Hospital alternative staging system for cutaneous squamous cell carcinoma: implications for clinical practice. J Am Acad Dermatol. 2019;80(1):106‐113.e2. 30003984 10.1016/j.jaad.2018.06.060

[22] Conde‐Ferreirós A , Corchete LA , Puebla‐Tornero L , et al. Definition of prognostic subgroups in the T3 stage of the eighth edition of the American Joint Committee on Cancer staging system for cutaneous squamous cell carcinoma: tentative T3 stage subclassification. J Am Acad Dermatol. 2020;85(5):1168‐1177. 32278798 10.1016/j.jaad.2020.03.088

[23] Fox M , Brown M , Golda N , et al. Nodal staging of high‐risk cutaneous squamous cell carcinoma. J Am Acad Dermatol. 2019;81(2):548‐557. 30227190 10.1016/j.jaad.2018.09.006 PMC8081061

[24] Maher JM , Schmults CD , Murad F , et al. Detection of subclinical disease with baseline and surveillance imaging in high‐risk cutaneous squamous cell carcinomas. J Am Acad Dermatol. 2020;82(4):920‐926. 31689446 10.1016/j.jaad.2019.10.067

