# Supplementary material for: Cutaneous squamous cell carcinoma staging may influence management in users: A survey study
Source: Cancer Med. 2021 Nov 18;11(1):94–103. doi: 10.1002/cam4.4426 (PMC8704160; doi:10.1002/cam4.4426)
Supplement: Supplementary file 1 — Fig S1 [file CAM4-11-94-s002.docx]

**Supplemental Figure 1: Cutaneous SCC Staging Survey**

| **What is your medical specialty?** |
| --- |
| Medical/Cutaneous Oncology Dermatologist  Mohs Surgeon  Medical Oncologist  Surgical Oncologist  ENT/Head and Neck Surgeon  Radiation Oncologist  Other: Write in response |
| **What region are you based and practicing in?** |
| Australia/New Zealand  United States  Canada  United Kingdom  Europe  Other: Write in response |
| **How many high-risk (however you define high-risk) CSCC cases have you seen in past 12 months?** |
| 0  1-10  11-25  26-50  >50 |
| **How many years have you been in practice (excluding residency)?** |
| 0-5 years  6-10 years  11-20 years  >20 years |
| **Which of the following best describes your practice environment? (Check all that apply)** |
| Academics  Private Practice  Veteran’s Affairs System  Closed Multi-Specialty System (i.e. Kaiser)  Other: Write in response |
| **Do you stage CSCC tumors that present or are referred to your practice?** |
| Yes  No |
| **If you do stage CSCC tumors, which tumors do you stage?** |
| All tumors  Suspected high-risk only  Other tumor subset: Write in response |
| **If you do stage CSCC tumors that present or are referred to your practice, do you record the stage in the patient's medical record or operative report?** |
| Yes  No |
| **If you do stage CSCC tumors, which staging systems do you utilize? (Check all that apply)** |
| AJCC 7th Edition (trunk and extremities only)  AJCC 8th Edition (head and neck)  UICC 8th Edition (all sites)  BWH Staging System  University Tubingen (Breuinger Risk System)  Other: Write in response |
| **If you do not stage CSCC tumors that present or are referred to your practice, please note why: (Check all that apply)** |
| T staging is not relevant to my practice or how I treat patients  I am not familiar with the available T staging systems  T staging takes too much time/resources  Other: Write in response |
| **If you utilize staging criteria to identify HRCSCC, which of the following stages do you consider as HRCSCC in AJCC 7th Edition system? Check all that apply** |
| T1  T2  T3  T4 |
| **If you utilize staging criteria to identify HRCSCC, which of the following stages do you consider as HRCSCC in AJCC 8th Edition system? Check all that apply** |
| T1  T2  T3  T4 |
| **If you utilize staging criteria to identify HRCSCC, which of the following stages do you consider as HRCSCC in UICC 8th Edition system? Check all that apply** |
| T1  T2  T3  T4 |
| **If you utilize staging criteria to identify HRCSCC, which of the following stages do you consider as HRCSCC in BWH staging system? Check all that apply** |
| T1  T2a  T2b  T3 |
| **If you utilize staging criteria to identify HRCSCC, which of the following stages do you consider as HRCSCC in the University Tubingen Risk system? Check all that apply** |
| Low Risk  High-risk |
| **Which of the following CSCC risk factors do you consider to be high-risk for poor outcomes? (Check all that apply)** |
| Clinical tumor diameter  Tumor depth  Perineural invasion  Histologic Differentiation  Patient Immunosuppression  Body site  Other: Write in response |
| **What tumor diameter do you consider to be high-risk for poor outcomes? (Check all that apply)** |
| Clinical tumor diameter >2cm  Other tumor diameter: Write in response |
| **What tumor depth do you consider to be high-risk for poor outcomes? (Check all that apply)** |
| Depth beyond subcutaneous fat  Depth measured >6mm from granular layer on H&E  Other tumor depth: Write in response |
| **What type of perineural invasion would you consider to be high-risk for poor outcomes? (Check all that apply)** |
| Any perineural invasion  Perineural invasion with clinical symptoms  Perineural invasion of a large caliber nerve (> 0.1mm diameter)  Perineural invasion of the deep dermis  Multifocal or multiple nerve perineural invasion |
| **What type of histologic differentiation would you consider to be high-risk for poor outcomes? (Check all that apply)** |
| Moderately differentiated histology  Poorly differentiated histology  Spindle cell histology  Desmoplasia on histology  Infiltrative histology |
| **What type of patient immunosuppression would you consider to be high-risk for poor outcomes? (Check all that apply)** |
| Stem Cell Transplant  Solid Organ Transplant  Hematologic malignancy  Chronic systemic disease requiring systemic immunosuppression or chronic prednisone (e.g. IBS, rheumatoid arthritis, etc.  Other: Write in response |
| **What body site would you consider to be high-risk for poor outcomes? (Check all that apply)** |
| Ear  Non-hair bearing (lipstick area/vermillion)  Cutaneous/ hair bearing lip  Other: Write in response |
| **What other risk factor(s) would you consider to be high-risk for poor outcomes?** |
| Write in response |
| **What is your preferred treatment option for HRCSCC?** |
| Mohs Micrographic Surgery or other technique to allow for 100% complete circumferential peripheral and deep margin assessment  Wide local excision with immediate closure and standard "bread loaf" permanent pathology  Wide local excision with delayed closure and standard permanent pathology  Wide local excision with intraoperative frozen section  Radiation monotherapy  Other: Write in response |
| **If you use pre or postoperative radiologic imaging to detect sub-clinical lymph node metastasis or distant metastasis in a patient with HRCSCC, what staging system do you use to determine whether or not to recommend imaging? (Check all that apply)** |
| AJCC 7th Edition (trunk and extremities only)  AJCC 8th Edition (head and neck)  UICC 8th Edition (all sites)  BWH Staging System  University Tubingen Risk System  I recommend radiologic imaging based on other formal criteria or guidelines  I recommend radiologic imaging, but not based on formal criteria or guidelines  I never recommend radiologic imaging |
| **For which AJCC 7th Edition system stage(s) do you consider radiologic imaging to detect subclinical lymph node metastasis or distant metastasis in a patient with HRCSCC (Check all that apply)** |
| T1  T2  T3  T4 |
| **For which AJCC 8th Edition system stage(s) do you consider radiologic imaging to detect subclinical lymph node metastasis or distant metastasis in a patient with HRCSCC (Check all that apply)** |
| T1  T2  T3  T4 |
| **For which UICC 8th Edition system stage(s) do you consider radiologic imaging to detect subclinical lymph node metastasis or distant metastasis in a patient with HRCSCC (Check all that apply)** |
| T1  T2  T3  T4 |
| **For which BWH Staging system stage(s) do you consider radiologic imaging to detect subclinical lymph node metastasis or distant metastasis in a patient with HRCSCC (Check all that apply)** |
| T1  T2a  T2b  T3 |
| **For which University Tubingen Point system stage(s) do you consider radiologic imaging to detect subclinical lymph node metastasis or distant metastasis in a patient with HRCSCC (Check all that apply)** |
| Low Risk  High-risk |
| **What formal criteria or guidelines do you use to recommend radiologic imaging?** |
| Write in response |
| **Please describe how you decide when to recommend radiologic imaging.** |
| Write in response |
| **If you recommend radiologic imaging, which imaging modality do you routinely obtain? (Check all that apply)** |
| CT scan  MRI  PET scan  Ultrasound  No preference |
| **If you use or recommend sentinel lymph node biopsy (SLNBx) to search for sub-clinical lymph node metastasis in a patient with HRCSCC, what staging system(s) do you use to determine whether or not to recommend SLNB? (Check all that apply)** |
| AJCC 7th Edition (trunk and extremities only)  AJCC 8th Edition (head and neck)  UICC 8th Edition (all sites)  BWH Staging System  University Tubingen Point System  I recommend SLNBx based on other formal criteria or guidelines  I recommend SLNBx, but not based on formal criteria or guidelines  I never recommend SLNBx |
| **For which AJCC 7th Edition system stage(s) do you consider sentinel lymph node biopsy (SLNBx) to detect subclinical lymph node metastasis or distant metastasis in a patient with HRCSCC (Check all that apply)** |
| T1  T2  T3  T4 |
| **For which AJCC 8th Edition system stage(s) do you consider sentinel lymph node biopsy (SLNBx) to detect subclinical lymph node metastasis or distant metastasis in a patient with HRCSCC (Check all that apply)** |
| T1  T2  T3  T4 |
| **For which UICC 8th Edition system stage(s) do you consider sentinel lymph node biopsy (SLNBx) to detect subclinical lymph node metastasis or distant metastasis in a patient with HRCSCC (Check all that apply)** |
| T1  T2  T3  T4 |
| **For which BWH Staging system stage(s) do you consider sentinel lymph node biopsy (SLNBx) to detect subclinical lymph node metastasis or distant metastasis in a patient with HRCSCC (Check all that apply)** |
| T1  T2a  T2b  T3 |
| **For which University of Tubingen Point system stage(s) do you consider sentinel lymph node biopsy (SLNBx) to detect subclinical lymph node metastasis or distant metastasis in a patient with HRCSCC (Check all that apply)** |
| Low Risk  High-risk |
| **What formal criteria or guidelines do you use to recommend SLNBx?** |
| Write in response |
| **Please describe how you decide when to recommend SLNBx.** |
| Write in response |
| **If you use or recommend post-operative adjuvant radiation therapy (ART) for a patient with HRCSCC, which staging system(s) do you use to recommend ART? (Check all that apply)** |
| AJCC 7th Edition (trunk and extremities only)  AJCC 8th Edition (head and neck)  UICC 8th Edition (all sites)  BWH Staging System  University Tubingen Point System  I recommend ART based on other formal criteria or guidelines  I recommend ART, but not based on formal criteria or guidelines  I never recommend ART |
| **For which AJCC 7th Edition system stage(s) do you consider adjuvant radiation therapy (ART) for patient with HRCSCC (Check all that apply)** |
| T1  T2  T3  T4 |
| **For which AJCC 7th Edition system stage(s) do you consider adjuvant radiation therapy (ART) for patient with HRCSCC (Check all that apply)** |
| T1  T2  T3  T4 |
| **For which AJCC 7th Edition system stage(s) do you consider adjuvant radiation therapy (ART) for patient with HRCSCC (Check all that apply)** |
| T1  T2  T3  T4 |
| **For which BWH Staging system stage(s) do you consider adjuvant radiation therapy (ART) for patient with HRCSCC (Check all that apply)** |
| T1  T2a  T2b  T3 |
| **For which University of Tubingen Risk system stage(s) do you consider adjuvant radiation therapy (ART) for patient with HRCSCC (Check all that apply)** |
| Low Risk  High-risk |
| **What formal criteria or guidelines do you use to recommend ART?** |
| Write in response |
| **Please describe how you decide when to recommend ART.** |
| Write in response |
| **Do you use or recommend post-operative adjuvant systemic therapy for a patient with HRCSCC?** |
| Yes  No |
| **If so, which adjuvant systemic therapy(s) do you employ (Check all that apply)** |
| Capecitabine  Systemic 5 fluorouracil  Platinum-based regimens  Anti-EGFR targeted molecular inhibitor  Anti-PD1/PDL-1 immunotherapy  I never use adjuvant systemic therapy  Other: Write in response |
| **If you use or recommend post-operative adjuvant systemic therapy for a patient with HRCSCC, which staging system do you use to recommend adjuvant systemic therapy? (Check all that apply)** |
| AJCC 7th Edition (trunk and extremities only)  AJCC 8th Edition (head and neck)  UICC 8th Edition (all sites)  BWH Staging System  University Tubingen Point System  I recommend adjuvant systemic therapy based on other formal criteria or guidelines  I recommend adjuvant systemic therapy, but not based on formal criteria or guidelines  I never recommend adjuvant systemic therapy |
| **For which AJCC 7th Edition system stage(s) do you consider post-operative adjuvant systemic therapy for patient with HRCSCC (Check all that apply)** |
| T1  T2  T3  T4 |
| **For which AJCC 8th Edition system stage(s) do you consider post-operative adjuvant systemic therapy for patient with HRCSCC (Check all that apply)** |
| T1  T2  T3  T4 |
| **For which UICC 8th Edition system stage(s) do you consider post-operative adjuvant systemic therapy for patient with HRCSCC (Check all that apply)** |
| T1  T2  T3  T4 |
| **For which BWH Staging system stage(s) do you consider post-operative adjuvant systemic therapy for patient with HRCSCC (Check all that apply)** |
| T1  T2a  T2b  T3 |
| **For which University Tubingen Point system stage(s) do you consider post-operative adjuvant systemic therapy for patient with HRCSCC (Check all that apply)** |
| Low Risk  High-risk |
| **What formal criteria or guidelines do you use to recommend adjuvant systemic therapy?** |
| Write in response |
| **Please describe how you decide when to recommend adjuvant systemic therapy.** |
| Write in response |
| **Do you ever recommend increased follow-up frequency for skin and lymph node evaluation for a patient with HRCSCC where clear surgical margins were obtained?** |
| Yes  No |
| **If you do recommend increased follow up, what frequency do you recommend for the first two years?** |
| Every 3 months  Every 4 months  Every 6 months  Other: Write in response |
| **If you do recommend increased follow-up frequency for skin and lymph node evaluation for a patient with HRCSCC, what system(s) do you use to recommend increased follow-up? (Check all that apply)** |
| AJCC 7th Edition (trunk and extremities only)  AJCC 8th Edition (head and neck)  UICC 8th Edition (all sites)  BWH Staging System  University Tubingen Point System  I recommend increased follow-up frequency based on other formal criteria or guidelines  I recommend increased follow-up frequency, but not based on formal criteria or guidelines  I never recommend increased follow-up frequency |
| **For which AJCC 7th Edition system stage(s) do you consider increased follow-up frequency for patient with HRCSCC (Check all that apply)** |
| T1  T2  T3  T4 |
| **For which AJCC 8th Edition system stage(s) do you consider increased follow-up frequency for patient with HRCSCC (Check all that apply)** |
| T1  T2  T3  T4 |
| **For which UICC 8th Edition system stage(s) do you consider increased follow-up frequency for patient with HRCSCC (Check all that apply)** |
| T1  T2  T3  T4 |
| **For which BWH Staging system stage(s) do you consider increased follow-up frequency for patient with HRCSCC (Check all that apply)** |
| T1  T2a  T2b  T3 |
| **For which University of Tubingen Point system stage(s) do you consider increased follow-up frequency for patient with HRCSCC (Check all that apply)** |
| Low Risk  High-risk |
| **What formal criteria or guidelines do you use to recommend increased follow-up frequency?** |
| Write in response |
| **Please describe how you decide when to recommend increased follow-up frequency.** |
| Write in response |
| **If you do recommend increased follow up frequency, do you follow up radiologically?** |
| Yes  No |
| **If you do follow up radiologically, what modality do you use? (Check all that apply)** |
| CT scan  MRI  PET scan  Ultrasound  No preference |
| **Please feel free to make any additional comments regarding this survey, the management of HRCSCC, or suggestions for future clinical trials.** |
| Write in response |
