# Supplementary material for: Cutaneous squamous cell carcinoma staging may influence management in users: A survey study
Source: Cancer Med. 2021 Nov 18;11(1):94–103. doi: 10.1002/cam4.4426 (PMC8704160; doi:10.1002/cam4.4426)
Supplement: Supplementary file 2 — Table S1 [file CAM4-11-94-s001.docx]

**Supplemental Table 1: Identification of Features as High-Risk by Survey Respondents**

|  | **Number of responses (% of each category)**  **N=156** | **Dermatologists**  **n=89** | **Other Cancer Specialists**  **N=67** | **P-Value** |
| --- | --- | --- | --- | --- |
| **Clinical Tumor Diameter** | 106 (68%) | 70 (79%) | 36 (54%) | **0.001** |
| >2 cm | 97 (62%) | 67 (75%) | 30 (45%) | **<0.001** |
| 2.1 -4 cm* | 5 (3%) | 1 (1%) | 4 (6%) | 0.151 |
| >4 cm* | 4 (3%) | 1 (1%) | 1 (1%) | 0.089 |
| Unspecified Criteria | 2 (1%) | 1 (1%) | 1 (1%) | 0.839 |
| **Tumor Depth** | 143 (92%) | 84 (94%) | 59 (88%) | 0.157 |
| Beyond subcutaneous fat | 117 (75%) | 70 (79%) | 47 (70%) | 0.225 |
| Measured >6mm from  granular layer | 89 (57%) | 56 (63%) | 33 (49%) | 0.088 |
| Other | 4 (3%) | 4 (4%) | 0 (0%) | 0.079 |
| **Perineural invasion (PNI)** | 154 (98%) | 88 (99%) | 66 (99%) | 0.839 |
| Any | 42 (27%) | 15 (17%) | 27 (40%) | **0.001** |
| PNI with clinical symptoms | 93 (60%) | 56 (63%) | 37 (55%) | 0.332 |
| PNI >0.1mm diameter | 126 (81%) | 82 (92%) | 44 (66%) | **<0.001** |
| PNI of the deep dermis | 51 (33%) | 28 (31%) | 23 (34%) | 0.706 |
| Multifocal or multiple PNI | 99 (63%) | 57 (64%) | 42 (63%) | 0.862 |
| **Histologic Differentiation/Pattern** | 132 (85%) | 88 (99%) | 44 (66%) | **<0.001** |
| Moderate differentiation | 22 (14%) | 20 (22%) | 2 (3%) | **0.001** |
| Poor differentiation | 131 (84%) | 87 (98%) | 44 (66%) | **<0.001** |
| Spindle cell | 93 (60%) | 63 (71%) | 30 (45%) | **0.001** |
| Desmoplasia | 58 (37%) | 40 (45%) | 18 (27%) | **0.021** |
| Infiltrative | 67 (43%) | 46 (52%) | 21 (3%) | **0.011** |
| Acantholytic* | 1 (1%) | 1 (1%) | 0 (0%) | 0.384 |
| **Immunosuppression** | 141 (90%) | 78 (88%) | 63 (94%) | 0.180 |
| Stem Cell Transplant | 94 (60%) | 47 (53%) | 47 (70%) | **0.029** |
| Solid Organ Transplant | 140 (90%) | 77 (87%) | 63 (94%) | 0.126 |
| Hematologic Malignancy | 113 (72%) | 68 (76%) | 45 (67%) | 0.201 |
| Chronic Systemic Disease | 89 (57%) | 41 (46%) | 48 (72%) | **0.001** |
| HIV* | 4 (3%) | 3 (3%) | 1 (1%) | 0.463 |
| Other Nonspecific | 4 (3%) | 1 (1%) | 3 (4%) | 0.315 |
| **Location** | 95 (61%) | 58 (65%) | 37 (55%) | 0.208 |
| Ear | 82 (53%) | 51 (57%) | 31 (46%) | 0.172 |
| Non-hair bearing lip | 79 (51%) | 56 (63%) | 23 (34%) | **<0.001** |
| Cutaneous / hair bearing lip | 27 (17%) | 11(12%) | 16 (24%) | 0.060 |
| Genitalia* | 2 (1%) | 1 (1%) | 1 (1%) | 0.839 |
| Temple/Mask Area Face* | 15 (10%) | 11(12%) | 4 (6%) | 0.180 |
| Scalp* | 14 (9%) | 14 (16%) | 0 (0%) | **0.001** |
| **Other*** |  |  |  |  |
| Lymphovascular Invasion | 3 (2%) | 3 (3%) | 0 | 0.129 |
| Nodal Metastasis at initial  tumor presentation | 2 (1%) | 0 (0%) | 2 (3%) | 0.101 |
| Recurrence | 9 (6%) | 5 (6%) | 4 (6%) | 0.926 |
| Scar/Radiation | 2 (1%) | 1 (1%) | 1 (1%) | 0.839 |

*written in by respondents

Bolded entries signify statistical significance
